# After a pair of self-control-intensive tasks, sucrose swishing improves subsequent working memory performance

**DOI:** 10.1186/2050-7283-1-22

**Published:** 2013-10-30

**Authors:** Evan C Carter, Michael E McCullough

**Affiliations:** Department of Ecology, Evolution and Behavior, University of Minnesota, St. Paul, MN 55108 USA; Department of Psychology, University of Miami, P.O. Box 248185, Coral Gables, FL 33124 USA

**Keywords:** Self-regulation, Self-control, Working memory, Ego depletion, Limited strength model of self-control, Learned industriousness, Carbohydrate mouthwash, Glucose swishing

## Abstract

**Background:**

The limited strength model of self-control predicts that acts of self-control impair subsequent performance on tasks that require self-control (i.e., “ego depletion”), and the majority of the published research on this topic is supportive of this prediction. Additional research suggests that this effect can be alleviated by manipulating participants’ motivation to perform—for instance, by having participants swish a drink containing carbohydrates, which is thought to function as a reward—or by requiring participants to complete two initial acts of self-control rather than only one.

**Methods:**

Here, we explore both the effect of having participants perform two initial tasks thought to require self-control (versus two less self-control-intensive tasks) and the effect of swishing a drink containing sucrose (compared to control drinks) on subsequent self-control. Outcomes were analyzed using standard null hypothesis significance testing techniques (e.g., analysis of variance, *t*-tests). In some cases, test statistics were transformed into Bayes factors to aid in interpretation (i.e., to allow for acceptance of the null hypothesis).

**Results:**

We found that performing two self-control-intensive tasks actually improved subsequent self-control when participants swished a drink containing sucrose between tasks. For participants who swished control drinks, we found no evidence of ego depletion.

**Conclusions:**

We conclude that claims that self-control failure is caused by the depletion of a resource (or that it functions as if it relies on a limited resource) merit greater circumspection. Our results—all of which were either null or contrary to predictions from the limited strength model—are important for researchers interested in patterns of self-control failure.

**Electronic supplementary material:**

The online version of this article (doi:10.1186/2050-7283-1-22) contains supplementary material, which is available to authorized users.

## Background

The limited strength model of self-control (Muraven & Baumeister, 2000) specifies that self-control draws on a finite “psychological (and physiological) resource” (Bauer & Baumeister, 2011; p. 79). Consequently, the model predicts that self-regulatory actions impair subsequent acts of self-control because they deplete the required resource, resulting in a state dubbed *ego depletion*. The so-called sequential task paradigm, in which participants first perform tasks to manipulate the exertion of self-control and then another task that enables measurement of any resulting reductions in self-control (which we refer to as *the depletion effect*), was designed to test this prediction (Baumeister et al., 1998). Many researchers who have used this paradigm report successful conceptual replication^a^ of the depletion effect (Hagger et al., 2010).

Some researchers have searched for the resource upon which self-control ostensibly draws. For example, Gailliot et al. (2007) proposed that acts of self-control deplete brain glucose levels and that ingesting sucrose (which contains glucose) forestalls ego depletion. However, Gailliot et al.’s (2007) findings have been questioned on the grounds of both theoretical plausibility and statistical robustness (Kurzban, 2010; Schimmack, 2012). Furthermore, the completion of self-control tasks of the kind that are typically used to test the depletion effect does not consistently lower blood glucose levels (Kurzban, 2010; Molden, et al., 2012), and published research suggests that the mere presence of sucrose in the mouth, which does not increase blood glucose (Molden, et al., 2012) eliminates the depletion effect (Molden, et al., 2012; Sanders et al. 2012; Hagger & Chatzisarantis, 2013). These findings suggest a motivational (i.e., glucose functions as a reward), rather than metabolic (i.e., glucose functions as fuel), explanation for the effect of glucose on depletion.

Other findings also suggest that the depletion effect can be eliminated by manipulating psychological variables such as motivation and expectations. For example, Muraven and Slessareva (2003) reported three experiments in which depletion was eliminated through motivation manipulations. Results from four other experiments suggest that the depletion effect obtains only when participants believe that self-control is limited, and that it disappears when participants do not expect to be depleted (Job et al. 2010; Martijn et al. 2002).

In the face of such evidence, which can be interpreted as contradictory to the limited strength model, (Vohs et al. 2012) have proposed that manipulations of belief or motivation can eliminate only *low* levels of ego depletion. To test this proposal, they ran two experiments using a modified sequential task paradigm. In the first experiment, they used (Job et al. 2010) methods to convince participants that willpower was either limited or unlimited. In the second experiment, participants’ motivation to perform was manipulated following Muraven and Slessareva’s (2003) methods (i.e., by manipulating the perceived importance of participants’ performance). Participants in both experiments then either completed a single control task, a single task requiring self-control, or a set of tasks requiring self-control. After the initial task (or set of tasks), participants completed two outcome tasks thought to require self-control. Vohs et al. (2012) predicted that completing more initial tasks would result in greater ego depletion, and that the manipulations of belief or motivation would only be effective at reducing less severe depletion (i.e., when participants had completed only one or two initial tasks rather than three or four). These patterns obtained, and were interpreted as evidence that “[a]cts of self-control and decision making do in fact deplete some energy resource” (Vohs, et al. 2012, p. 4).

Vohs et al.’s (2012) conclusion that an energy resource had been depleted is problematic for two reasons. First, terms like “resource” and “strength” can be read as purely metaphorical (rather than literal) because the resource in question has never been measured (nor has any means of measuring it, other than via blood glucose, been proposed). Second, Vohs et al.’s (2012) interpretation relies on the assumption that a greater number of initial tasks should result in a more severe performance decrement (since more of the resource has been used). However, several previous experiments revealed that including more than a single task in the initial phase of the sequential task paradigm actually *increases* performance on a subsequent task: Converse and DeShon (2009), for instance, reported three experiments in which completing two initial self-control-intensive tasks (rather than two initial tasks requiring less self-control) improved performance on subsequent self-control tasks. Furthermore, the literature on learned industriousness (which inspired [Converse and DeShon 2009work) includes many experiments that use a paradigm that is nearly identical to the sequential task paradigm. These experiments tend to show that requiring greater initial outlays of effort (e.g., on math problems and anagrams, which are thought to require self-control) causes better performance on a final task—usually of the kind that is thought to require self-control (e.g., analytical writing; Eisenberger et al. 1982; Hagger et al. 2010).

In light of the literature on learned industriousness (see Eisenberger 1992), Converse and DeShon’s (2009) findings, and recent experiments on tasting (rather than digesting) glucose (e.g., Molden et al. 2012; Sanders et al. 2012; Hagger & Chatzisarantis 2013), the common interpretation of the depletion effect—that low performance is due to low resources—seems far from adequate. Therefore, we designed the current study to examine two issues: (a) whether the depletion effect obtains when more than one task is used during the “depletion” phase (i.e., before subsequent tasks that serve as dependent variables); and (b) whether the depletion effect, when induced by multiple initial tasks, can indeed be reduced by having participants swish a drink sweetened with sucrose compared to a drink sweetened with a control sweetener (sucralose) or an unsweetened drink. We reasoned that different patterns of results would be consistent with specific, previously proposed models: Based on the limited strength model (Baumeister, et al. 1998; Vohs, et al. et al. 2012), one would predict that participants who complete two initial self-control tasks should perform worse on a third self-control task compared to participants who complete two initial tasks that are relatively less self-control-intensive (i.e., the depletion effect). Based on the work by Gailliot et al. (2007), one would also predict that the depletion effect would not be observed for participants who have *ingested* glucose. However, based on more recent work (Molden et al. 2012; Sanders et al. 2012; Hagger & Chatzisarantis 2013), one would predict that the depletion effect should also be reduced for participants who merely rinsed their mouths with a drink containing glucose, not necessarily only those that ingested glucose.

In contrast, based on experiments inspired by learned industriousness, one should predict that completing two self-control-intensive tasks (i.e., expending a relatively higher amount of effort) should actually increase subsequent self-control performance (e.g., Eisenberger et al. 1982; Eisenberger 1992; Converse & DeShon 2009). The mechanism thought to underlie findings in the learned industriousness work is described by the secondary reward theory of industriousness: Rewarding high effort results in continued high effort because the sensation of effort is learned as a predictor of reward (Eisenberger 1992). Based on the secondary reward theory of industriousness, therefore, one would predict that increased self-control performance on a third task will only follow the completion of self-control-intensive tasks if participants are subsequently rewarded in some way. In the current experiment, the sweet taste of either sucrose- or sucralose-sweetened drinks may be rewarding, so based on the secondary reward theory of industriousness, one would predict that participants who swish either sucrose- or sucralose-sweetened drinks following high effort (i.e., completing two self-control tasks, rather than two relatively less self-control-intensive tasks) will perform better on the third self-control task. Note, however, that Converse and DeShon (2009) found that, for participants who completed multiple, unrewarded self-control tasks, subsequent self-control performance was improved relative to the performance of participants who completed multiple, unrewarded tasks that required relatively less self-control. Based on these findings, one would predict that completing multiple initial self-control-intensive tasks should increase subsequent performance, regardless of whether completion of these tasks was rewarded (i.e., regardless of the type of drink given to participants).

## Methods

### Participants

Upon arriving at the laboratory, participants read and signed a consent form that had been reviewed and approved by the University of Miami Institutional Review Board. All methods and procedures were likewise approved by the University of Miami Institutional Review Board. Participants (N *=* 257) completed the experiment during individual sessions in exchange for $10 and partial fulfillment of a course requirement. We instructed participants to avoid eating for ≥ 3hours before attending the laboratory session. Seventeen participants were excluded from data analysis because they failed to follow instructions (e.g., failed to fast before the experiment). Five additional participants’ dependent variable measurements were lost due to experimenter error. Therefore, the final sample included 235 participants (110 males).

We planned to restrict data collection to a single semester. We examined the data at several points before the semester’s end, and approximately 60% of the way through data collection, no effects had reached statistical significance, so we stopped assigning participants to the unsweetened rinse condition to increase power for other comparisons that we viewed as more important. Consequently, the *n*s of the two sweetened rinse conditions (*n*_sucrose_ = 92, *n*_sucralose_ = 93) are larger than is the *n* for the unsweetened condition (*n*_unsweetened_ = 50). Testing predictions prior to the completion of data collection increases the risk of false positives, though this risk drops as sample sizes increase (Simmons et al. 2011), and the end of data collection was not determined by any particular pattern of results.

### Procedure

Participants completed the experiment individually during one-hour laboratory sessions that we had described as investigating “impression formation and cognitive function”. Participants were randomly assigned to either a high-effort or a low-effort condition (see below) and one of three rinse conditions: rinsing with a Kool-aid drink that was either unsweetened, sweetened with sucrose (171grams of sugar dissolved into 2 quarts of Kool-aid), or sweetened with sucralose (14 tablespoons of Splenda dissolved into 2 quarts of Kool-aid). Note that the Kool-aid flavoring mix we used was not sweet by itself.

Participants first completed two commonly used depletion tasks: (a) watching a brief video of a woman being interviewed during which words are presented in the bottom of the screen (Schmeichel et al. 2003); and (b) writing an essay describing a vacation (Schmeichel 2007). Participants in the high-effort condition were instructed to avoid reading the words on the screen during the video and to avoid using the letters “a” and “n” while writing the essay. Participants in the low-effort condition were instructed to watch the video as they would watch any other video and received no additional instructions for the essay.

Following these two initial tasks, participants were told that they would be participating in a “taste test” during which they would taste (but not swallow) and rate a drink. Each participant was given six ounces of the appropriate drink and instructed to take a sip of the drink, swish it for ten seconds, and then spit into another cup. Participants were asked to repeat this process until they had tasted the full six ounces. In previous work using this method, compliance with the instructions not to swallow the drink was assessed through the measurement of blood glucose (Molden, et al. 2012). Results indicated that even if participants had ingested a small amount of glucose, contrary to investigators’ instructions, it was insufficient to increase blood glucose.

Following Gailliot et al. (2007), ten minutes elapsed between completion of the taste test and the beginning of the dependent variable measurements. During this time, participants completed a questionnaire that included rating items about the tasks and the drink they sampled, the Brief Mood Introspection Scale (BMIS; Mayer & Gaschke 1988), and some additional items not analyzed here (see Additional files 1 and 2). Afterward, participants sat quietly for the balance of ten minutes, ostensibly waiting for the experimenter to prepare the next part of the experiment.

Next, participants completed a version of a working memory task called the operation span (OSPAN), in which they were presented with sets of words to remember. Participants were presented with 15 sets of words, containing between two and five words. In each set, words were presented one at a time, and the presentation of a word was followed by the presentation of a mathematical equality, such as *(9 x 3) – 1 = 2*. Participants were instructed to remember each word until the end of a set, at which point they were asked to recall as many words as possible from only the set they had just completed. Additionally, participants were instructed that when they saw an equality, they were to respond either “yes” or “no” to indicate whether they believed the equality was true. The rate of presentation of word/equality pairs was controlled by the participant. The OSPAN provides four possible measures of working memory performance: the total number of full sets of words remembered (maximum 15 sets), the total number of words remembered across all sets (maximum 48 words), the longest set of words remembered (maximum five words), and the number of words in fully recalled sets only (maximum 48 words). Schmeichel (2007) reported that OSPAN performance, as measured by each of the above variables, generally decreased for participants who had previously exercised self-control (i.e., the depletion effect).

After the OSPAN, participants completed another questionnaire comprising two items about how difficult and how boring they found the OSPAN, an item about the last time they had eaten, and several items irrelevant to the present work (see Additional file 1). Participants were then thanked, debriefed, and paid.

## Results

Dependent variables were analyzed using 2 (effort: high vs. low) × 3 (rinse: sucrose, sucralose, or unsweetened) analysis of variance (ANOVA). See Table 1 for all test statistics for these models; see Additional file 3, for *n*s, means, and standard deviations. For between-condition comparisons, we used independent-samples *t*-tests when either the main effect for rinse or the effort*rinse interaction reached statistical significance (See Table 2).

**Table 1:** **Full factorial ANOVA results for the four experiment outcome categories**

Outcome		Main effects		Interaction effect
		Effort	Rinse	Effort × rinse
Rinse rating:	Sweetness	*F*(1, 229) = 1.26	*F*(2, 229) = 40.58***	*F*(2, 229) = 1.28
	Pleasantness	*F*(1, 229) = 0.83	*F*(2, 229) = 16.20***	*F*(2, 229) = 1.91
	Liking	*F*(1, 228) = 0.00	*F*(2, 228) = 17.23***	*F*(2, 228) = 1.63
Initial task	Video difficulty	*F*(1, 229) = 1.32	*F*(2, 229) = 4.07*	*F*(2, 229) = 0.15
	Essay difficulty	*F*(1, 229) = 412.00***	*F*(2, 229) = 0.58	*F*(2, 229) = 0.22
Mood:	Pleasant-unpleasant	*F*(1, 225) = 0.30	*F*(2, 225) = 0.30	*F*(2, 225) = 0.05
	Active-calm	*F*(1, 225) = 0.40	*F*(2, 225) = 0.85	*F*(2, 225) = 1.52
OSPAN:	Difficulty	*F*(1, 212) = 4.20*	*F*(2, 212) = 3.69*	*F*(2, 212) = 1.39
	Boredom	*F*(1, 212) = 0.35	*F*(2, 212) = 1.50	*F*(2, 212) = 0.61
	Performance	*F*(1, 229) = 5.86*	*F*(2, 229) = 0.08	*F*(2, 229) = 3.54*

*Note.* ***p < .001; *p < .05.

**Table 2:** **Post-hoc mean comparisons and tests of simple effects for rinse ratings, initial task ratings, and OSPAN ratings and performance**

Rinse ratings:	Initial task difficulty ratings	OSPAN ratings and performance
Liking	Video difficulty	OSPAN difficulty
*t*_*un-s*_ *(140) = -5.94, p < .001, d = -1.04*	***t***_**un-s**_ **(141) = -2.81,** ***p =*** **.006,** ***d*** **= -.49**	^†^ *t*_un-s_(112.55) = -2.09, *p* = .04, *d* = .38
^†^ ***t***_**un-su**_ **(119.38) = -5.22,** ***p*** **< .001,** ***d*** **= -.86**	*t*_un-su_(140) = -1.29, *p =* .20, *d* = -.22	^†^ *t*_un-su_(118.18) = -.02, *p =* .98, *d* = -.01
*t*_s-su_(182) = .91, *p* = .37, *d* = .13	*t*_s-su_(183) = -1.80, *p* = .07, *d* = .27	*t*_s-su_(171) = -2.27, *p* = .03, *d* = -.35
Sweetness		OSPAN performance
^†^ ***t***_**un-s**_ **(67.49) = -6.70,** ***p*** **< .001,** ***d*** **= -1.36**		*t*_un: hi-lo_(48) = -1.76, *p* = .09. d = .50
^†^ ***t***_**un-su**_ **(69.97) = -6.48,** ***p*** **< .001,** ***d =*** **-1.30**		*t*_su: hi-lo_(90) = -.59, *p =* .56, *d* = -.12
*t*_s-su_(183) = .26, *p* = .79, *d =* .04		***t***_**s: hi-lo**_ **(91) = -3.05,** ***p =*** **.003,** ***d*** **= .63**
Unpleasantness		
^†^ ***t***_**un-s**_ **(128.8) = 6.58,** ***p*** **< .001,** ***d*** **= 1.05**		
^†^ ***t***_**un-su**_ **(133.66) = 5.01,** ***p*** **< .001,** ***d*** **= .79**		
*t*_s-su_(183) = -1.29, *p* = .20*, d* = -.19		

*Note.* Alpha levels have been corrected using a Bonferroni correction within each outcome category (i.e., each column), such that *alpha* = .006 for rinse ratings, .017 for initial task difficulty ratings, and .008 for OSPAN ratings and performance. Italicized font indicates statistical significance relative to the corrected alpha level. ^†^Indicates that equal variances between groups has not been assumed (based on a statistically significant Levene's test). “un” = Unsweet rinse condition. “s” = Sucrose rinse condition. “su” = Sucralose rinse condition. “hi” = High-effort condition and “lo” = Low-effort condition. Note that in some cases, data for each participant were not always available; see Additional file 3, for *n*s, means, and standard deviations.

For each of the main effects and interactions in the ANOVAs, *alpha* = .05. However, the *alpha* levels for follow-up tests were modified using a Bonferroni correction specific to outcome category (i.e., the category-wise error rate was held constant). There are four main categories of outcome variables: Rinse ratings, initial task ratings, self-reported mood, and OSPAN ratings/performance (see Tables 1 and 2).

### Rinse, initial task, and mood ratings

Participants reported disliking the unsweetened rinse more than either of the sweetened rinses. They also rated the unsweetened rinse as more unpleasant and less sweet than either of the sweetened rinses (ratings for the two sweetened rinses did not differ; see Table 2, column 1). For initial task ratings, participants in the high-effort condition reported that they found the essay more difficult than did participants in the low-effort condition. Additionally, participants in the sucrose rinse condition reported that the video task was more difficult than did participants in the unsweetened rinse condition (ratings were not different between the sucralose and unsweetened conditions; see Table 2, column 2). For models predicting the mood rating scores, no terms reached statistical significance (see Table 1).

### OSPAN ratings and performance

The effort and rinse conditions did not affect how boring participants found the OSPAN. For the difficulty ratings of the OSPAN, the main effect for effort condition was statistically significant: Participants in the high-effort condition rated the OSPAN as subjectively less difficult than did those in the low-effort condition. The main effect for rinse condition was also significant, but post-hoc tests did not reveal any statistically significant pairwise differences.

As mentioned above, the OSPAN (Schmeichel 2007) yields four measures of working memory capacity. These measures were highly intercorrelated (all *r*s ≥ .75; see Additional file 4). To condense the number of statistical tests required, we used principal components analysis to reduce the four potential outcome variables to a single score that reflected variation in OSPAN performance^b^. One clear principal component emerged (Eigenvalue of 3.55), which accounted for 88.84% of the variance in participants’ scores on the four measures of working memory capacity. Participants’ scores on this component served as our primary outcome variable for testing changes in OSPAN performance.

**Figure 1:**
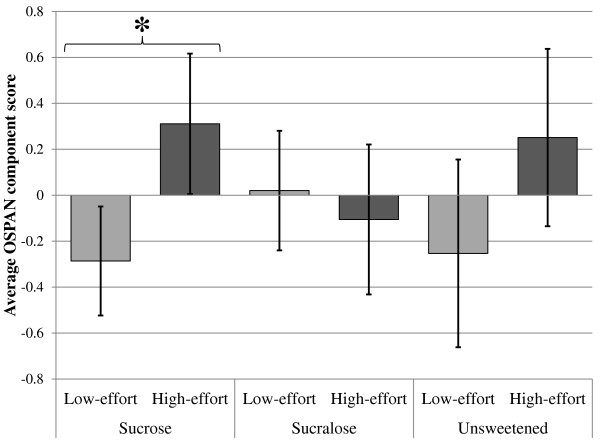
**Average OSPAN performance as a function of effort and rinse conditions.** Note.*p = .003. Error bars are equal to 95% confidence intervals.

For the OSPAN component score, the main effect for rinse was nonsignificant, but the main effect for effort was statistically significant: Overall, participants in the low-effort condition had *worse* (not better) working memory performance than did participants in the high-effort condition (*d* = -0.30). This main effect was modified by a significant effort-by-rinse interaction. We decomposed this interaction by comparing the means of the high-effort group and the low-effort group at each level of the rinse factor (see Table 2, column 3). Participants in the low-effort condition did not perform differently from those in the high-effort condition if they had previously swished an unsweetened (*d* = 0.50) or sucralose-sweetened rinse (*d* = -0.12), but participants in the high-effort condition performed significantly better than those in the low-effort condition if they had previously swished the sucrose-sweetened rinse (*d* = 0.63; see Figure 1 and Table 2). These data resist easy interpretation in terms of the limited strength model, but are reasonably consistent with the notion of learned industriousness inasmuch as the presence of an unconditioned reinforcer (the taste of sugar) apparently increased subsequent mental effort.

### Follow-up analyses of OSPAN performance using Bayes factors

The above results were obtained using standard statistical methodology (i.e., null hypothesis significance testing), which is both biased toward rejecting the null hypothesis (particularly when the sample size is large) and unsuitable for quantifying evidence for the null hypothesis (i.e., failure to reject the null can only be interpreted as a state of ignorance; Rouder et al. 2009). Given the importance of null findings for advancing theory (Laws 2013), we conducted follow-up analyses that do not suffer from the above limitations by calculating Bayes factors for independent-samples *t*-tests (Rouder et al. 2009). Using a web-based application (http://pcl.missouri.edu/bayesfactor), we calculated Bayes factors for the three independent samples *t*-tests that were used to compare OSPAN performance between the high- and low-effort groups for each of the three rinse conditions (i.e., the *t*s in the second row, third column of Table 2). These Bayes factors can be thought of as ratios of the evidence for the null hypothesis to evidence for the alternative hypothesis (i.e., Bayes factors smaller than 1 represent support for the alternative, whereas Bayes factors greater than 1 represent support for the null). Bayes factors can be directly interpreted (e.g., a Bayes factor of 6 means that the null is six times more likely than the alternative). For the Bayes factor calculation we used, the null hypothesis is specified as a difference of zero between means of the groups, and the alternative hypothesis is specified as any non-zero difference between groups (as it is in the standard *t*-test).

The following Bayes factors were found across levels of the rinse factor. In the sucrose condition, the Bayes factor was .096, which can be interpreted as “substantial evidence” that for people who have tasted sucrose, a high outlay of initial cognitive effort led to better performance on the OSPAN (rather than worse performance, as the limited strength model would predict). In the sucralose condition, the Bayes factor was 5.31, which can be interpreted as “substantial evidence” that, for people who have tasted sucralose, pairs of high-effort or low-effort initial tasks do not produce differences in subsequent OSPAN performance (i.e., the difference between performance in the two groups is zero). In the unsweetened rinse condition, the Bayes factor was 1.22; which can be interpreted as merely “anecdotal evidence” (i.e., “worthy of no more than a bare mention”; Wagenmakers et al. 2011) in favor of the null hypothesis.

## Discussion

Our results suggest that the presence of sucrose in the mouth (but, importantly, not lower in the digestive tract; Gailliot et al. 2007) does not merely return performance to normal levels, as observed previously (e.g., Molden et al. 2012), but instead may enhance performance following high mental effort. This finding is generally consistent with all motivation-based accounts of performance, in which high self-control performance is theorized as being due to a strategic increase in effort designed to achieve tasks that have been deemed important or that result in the receipt of reward (e.g., Eisenberger 1992; Beedie & Lane 2012; Baumeister & Vohs 2007; Molden et al. 2012; Inzlicht & Schmeichel 2012; Kurzban et al. (2013)). Importantly, however, the lack of evidence for the depletion effect (or, in terms of the Bayesian analysis, the evidence for the null model), makes our results difficult to reconcile with all models that predict decreased self-control performance as a function of previous self-control (e.g., Beedie & Lane 2012; Baumeister & Vohs 2007; Molden et al. 2012; Inzlicht & Schmeichel 2012; Kurzban et al. (2013)).

Instead, our data seem most consistent with an interpretation based on the secondary reward theory of industriousness (Eisenberger 1992), which does not predict that initial high effort will necessarily lead to subsequent low effort. For example, the taste of sucrose after the initial effort required in the high-effort condition may have reinforced high mental effort so that on the subsequent working memory task, participants worked harder and performed better, whereas for participants in the low-effort condition, the taste of sucrose encouraged continued low-levels of effort. Interestingly, if this interpretation is correct, it would appear that the sweet taste of sucralose did not function as a reward, which would be consistent with previous work showing that, in humans, the presence of carbohydrates in the mouth is related to patterns of activation in brain regions that are typically associated with the receipt of reward, whereas the presence of saccharin, an artificial sweetener, is not (Chambers et al. 2009). Of course, we offer this interpretation *post hoc*, and the experiment reported here was exploratory, so we caution against overconfidence in this explanation. Our findings that sucrose in the mouth improves performance following high mental effort should serve to motivate future replication efforts, rather than as solid evidence that such a phenomenon exists.

Nevertheless, because performing high-effort initial tasks rather than low-effort initial tasks did not *reduce* performance in any of the rinse conditions, our findings represent a failed conceptual replication of the depletion effect, as predicted by the limited strength model (e.g., Baumeister, et al. 1998; Gailliot et al. 2007; Hagger et al. 2010; Vohs et al. 2012). The published literature evaluating the depletion effect contains very few contradictory results such as ours (e.g., 196 of the 198 effect sizes included in Hagger et al.’s (2010) meta-analysis were in the direction predicted by the limited strength model, and only 47 were statistically non-significant), but the relatively large size of our sample (*contra*; Hagger et al. 2010) leads us to think that the present results should be taken seriously by researchers interested in self-control. Importantly, the fact that the relatively large experiment reported here yielded a clear lack of support for the depletion effect is consistent with concerns we have raised elsewhere that the current meta-analytic evidence for the depletion effect may be caused by publication bias, and that the true underlying effect size may be either small or no different from zero (Carter & McCullough [Bibr CR6][Bibr CR7]).

Given the results we report here, as well as our other work in this area (Carter & McCullough 2013a, 2013b), it seems plausible that the depletion effect, as measured by the sequential task paradigm, may not be a robust empirical phenomenon. An interpretation that is more favorable to the limited strength model might be that the sequential task paradigm is not an appropriate experimental procedure for studying the effect of previous acts of self-control on subsequent self-control performance and perhaps different experimental procedures, such as those used in the literature on cognitive fatigue (see Ackerman 2011), may measure a real phenomenon that is conceptually similar to the depletion effect. An even more favorable interpretation (albeit, one that ignores the meta-analytic conclusions that we have reported elsewhere; Carter & McCullough 2013) might be that the depletion effect is moderated by the type of experimental task used in the sequential task paradigm—that is, contrary to what was shown by Schmeichel (2007) perhaps OSPAN performance does not decrease when participants are depleted, but performance on other outcome tasks, such as persistence at difficult tasks, does (e.g., Baumeister, et al. 1998). It is noteworthy that the OSPAN is not especially widely used in the literature on the limited strength model (Hagger et al. 2010)^c^. However, according to the limited strength model, performance on any task that is thought to require self-control, such as the OSPAN, should suffer as a function of previous acts of self-control, so if it is true that the depletion effect is moderated by task type, the limited strength model will require revision on the basis of the results we have reported here.

The lack of a method for directly measuring the resource on which self-control relies means that resource-based explanations can be made consistent with the pattern of data we report here: For example, one might propose that the depletion effect would have been observed in the present experiment if participants had been required to complete a third initial task (i.e., our participants were simply not fully depleted; Vohs et al. 2012). One might also argue that participants who performed well on the OSPAN used their remaining resources to do so, and their depleted state would have been revealed had we included one more dependent variable. It will only be possible to rule such speculations out after the resource underlying self-control has been identified and a method for measuring it developed. Of course, a similar criticism can be leveled at any motivation-based explanation for self-control failure that is not sufficiently specific about the relationship between motivation and self-control. Thus, future work by theorists interested in resource-based and motivation-based explanations of self-control failure, such as the limited strength model, should focus on identifying and directly measuring the resource in question, or the process by which motivation changes (e.g., as proposed by Kurzban et al. (2013), the motivation to perform on a task is a function of opportunity cost: The greater the potential rewards the participant forgoes by putting effort into the task, the lower the participant’s motivation to perform the task).

One important limitation of the current study is that we did not measure blood glucose, so we cannot be certain that swishing the glucose sweetened drink did not affect blood glucose levels; that is, it is possible that some participants swallowed some of the glucose that they were asked to swish. However, given the results of previous work that suggests that swishing procedures that are almost identical to those we used here do not affect blood glucose levels (Molden et al. 2012, Experiment 4), it seems likely that our procedures also did not increase blood glucose. Furthermore, even if participants did ingest some portion of the drinks they were given, our major findings still present problems for the limited strength model because we found no evidence for a decrease in self-control performance following the completion of tasks that required self-control. Consequently, our tentative explanation for the results we did obtain, which rely on the concept of learned industriousness, would still hold (i.e., the presence of glucose in the mouth should function as a reward, rather than as the replenishment of a resource, just as its ingestion should, though perhaps with weaker effect). Nevertheless, future experimenters might consider measuring blood glucose to better arbitrate between the effects of sensing glucose in the mouth rather than in the digestive system.

A second limitation of the current work is the possibility that our null findings were the result of inadequate power. We did not conduct an *a priori* power analysis for our tests of the depletion effect (as mentioned, our data collection plan was to collect as much as possible in one semester). *A priori* power analyses are difficult to conduct for conceptual replications because it is not known if the parameter estimates provided by previous work generalize to the procedures that constitute the conceptual replication. Nevertheless, assuming the alternative hypothesis is true (i.e., the depletion effect is non-zero) for participants in the sucralose-sweetened and unsweetened rinse conditions, then our test of the depletion effect would have had 80% power for effect sizes of *d* = 0.47 or greater. According to Hagger et al. (2010), who provided a variety of meta-analytic estimates of the depletion effect for subsamples of experiments that were methodologically similar to ours, the depletion effect is at least this large.

However, if the depletion effect is nonzero but considerably smaller than *d* = 0.47, then the tests we conducted here are underpowered, and it is possible that our failure to find evidence for the depletion effect was due to low statistical power. According to one interpretation of our re-analyses of Hagger et al.’s (2010) meta-analytic data (Carter & McCullough 2013a, 2013b), it is possible that the depletion effect is indeed nonzero, but smaller than was originally estimated. Specifically, we found that based on one method of correcting for the influence of publication bias (Moreno et al. 2009), it is possible that the depletion effect is *d* = 0.25. If this estimate is correct, then any test that comprises fewer than 252 participants *per group* will have less than 80% power. Importantly, 188 of the 198 experiments reviewed by Hagger et al. (2010) had a *total* sample size of *N* = 100 or less, and the two largest experiments had total sample sizes of *N* = 284 and 501. In other words, if the depletion effect is some small, nonzero magnitude, then it would appear to be the case that the vast majority of experiments that have been conducted have been underpowered, including the one we report here.

Based on the experiment described here, as well as our re-analysis of Hagger et al.’s (2010) work, we believe that the balance of the evidence supports the conclusion that the depletion effect is either not a robust phenomenon or that it is considerably smaller than has been previously reported. This conclusion is directly contrary to those that have been drawn by some other researchers (e.g., Vohs et al. 2012; Hagger et al. 2010). Thus, as we have recommended elsewhere (Carter & McCullough 2013a, 2013b), we believe that it is critical that researchers conduct large-scale direct replications of the classic tests of the depletion effect (e.g., replications of the experiments reported by [Baumeister et al. 1998, but with total samples of at least *N* = 504).

## Conclusions

Our findings, when combined with other recently obtained results, cast doubt on the generality of the depletion effect (e.g., Converse & DeShon 2009; Carter & McCullough 2013a, 2013b), and on the role of glucose as the limited resource underlying self-control (e.g., Molden et al. 2012). Collectively, this work implies that the proposition that self-control relies on an actual resource (or even functions as if it did) requires additional empirical and theoretical attention before scientists should swallow it whole.

## Endnotes

^a^Following Schmidt (2009), we use the term conceptual replication to refer to any attempt at replicating a previous test via different methods. Conceptual replication can be contrasted with direct replication, which is a repetition of a previous test via identical methods (Schmidt 2009). For a test of the depletion effect, most experiments are conceptual replications because different combinations of self-control tasks are used in the sequential task paradigm.

^b^Principal component analysis, or PCA, is a mathematical transformation that allows researchers to reduce a set of variables to one or more so-called components. PCA is technically different from factor analysis methods, which are based on the common factor model and should be used when the researcher wishes to explore how unobserved latent variables (i.e., factors) underlie the correlations between measured variables. Factor analysis, therefore, is based on a specific model and tests a specific hypothesis. PCA, on the other hand, should be used when a researcher simply wishes to reduce a set of measures down to a set of independent components that account for as much of the variance between the observed variables as possible (Fabrigar et al 1999). We chose to use PCA because we were concerned with how our experimental manipulations would affect variance in OSPAN performance scores, rather than any hypothesis about the latent variables underlying OSPAN performance.

^c^We choose to use the OSPAN in our experiment because it has been argued that working memory performance indexes something fundamental to self-control (e.g., Hofmann et al. 2011).

## Electronic supplementary material

Additional file 1: **Additional methods.** (DOCX 20 KB)

Additional file 2: Table S2: Results for the three supplemental analysis outcome categories. (DOCX 16 KB)

Additional file 3: Table S1: Cell means, standard deviations, and sample sizes for each combination of effort and rinse conditions. (DOCX 26 KB)

Additional file 4: Table S3: Intercorrelations for measures of OSPAN. (DOCX 14 KB)
