# Supplementary material for: After a pair of self-control-intensive tasks, sucrose swishing improves subsequent working memory performance
Source: BMC Psychol. 2013 Oct 30;1(1):22. doi: 10.1186/2050-7283-1-22 (PMC4269986; doi:10.1186/2050-7283-1-22)
Supplement: Supplementary file 1 — Additional file 1: Additional methods. (DOCX 20 KB) [file 40359_2013_19_MOESM1_ESM.docx]

After a Pair of Self-Control-Intensive Tasks,

Sucrose Swishing Improves Subsequent Working Memory Performance

Evan C. Carter and Michael E. McCullough

**Supplementary methods:**

**Additional questionnaire items.** Participants completed several questionnaire items in addition to those described in the main text. On the first questionnaire, which followed the “taste test,” participants were asked to provide estimates of the amount of time they had spent completing each of the initial tasks, how often they looked at the words during the attention video (on a scale from 1, *never looked*, to 9, *looked at every word*), whether they would like to drink the drink they had tasted on a regular basis, and the extent to which they described their present mood as bored, restless, distracted, fatigued, engaged, or curious (on a scale of 1, *definitely do not feel*, to 4, *definitely feel*). On the second questionnaire, which followed the OSPAN, participants were asked to provide an estimate of the amount of time they had spent completing the OSPAN as well as some additional items (i.e., demographic information, Future Time Orientation Scale; Gjesme, 1979) that were not analyzed.

**Supplementary results and discussion**

As in the main text, unless otherwise noted, variables were analyzed using a 2 (effort: high vs. low) x 3 (rinse: sucrose, sucralose, or unsweetened) analysis of variance (ANOVA). As with the primary analyses, *alpha =* .05 for ANOVA models, but *alpha* levels for follow-up tests were adjusted (according to a Bonferoni correction) for each category of dependent variable. Three categories were analyzed: Time estimates, additional task rating, and additional mood ratings.

Cell means, standard deviations, and sample sizes are presented in Table S1 for all variables that were analyzed. Test statistics are presented in Table S2. Follow-up tests are reported in-text. Intercorrelations for measures of OSPAN performance are presented in Table S3.

**Time estimates.** To create an index of time estimation accuracy, participants’ estimates of the time that was spent watching the video, writing the essay, and completing the OSPAN were subtracted from the actual time taken during these intervals. No differences were found for estimates of time spent writing the essay or completing the OSPAN (see Table S2). In contrast, a statistically significant main effect for effort condition emerged for estimates of time spent watching the video; however, the follow-up test was non-significant, *t*(227) = 1.83, *p* = .07, *d* = .24.

These results represent a non-replication of the findings presented by Vohs and Schmeichel (2003), who found that performing self-control-intensive tasks alters the subjective experience of time, and that this alteration mediates subsequent self-control failure.

**Additional task rating.** A statistically significant main effect for effort condition was found for the extent to which participants reported looking at the words on the bottom of the screen during the video task (see Table S2). A follow-up test indicated that participants who were instructed not to look at the words during the video task reported looking at the words far fewer times, *t*(233) = -26.42, *p* < .000, *d* = -3.45.

A multiple logistic regression model was run to assess whether effort or rinse condition (or the interaction of the two) affected participants’ desire to drink they tasted during the taste test on a regular basis. Dummy codes were created for effort (the low-effort condition was coded as 0), for the sucrose-sweetened rinse condition, and the sucralose-sweetened rinse condition (the unsweetened rinse condition was coded as 0 for both of these variables). Two interaction terms (effort*sucrose and effort*sucralose) were created after centering the effort, sucrose, and sucralose dummy codes. In this model, both the terms for sucrose and for sucralose were statistically significant, such that participants in the sucrose-sweetened rinse condition and the sucralose-sweetened rinse condition were approximately thirteen (OR = 13.90) and seven (OR = 7.70) times, respectively, more likely to report that they would want to regularly drink the drink they had tasted compared to participants in the unsweetened-rinse condition (see Table S2). These results are consistent with the results regarding rinse ratings reported in the main text.

**Additional mood ratings.** Neither the main effects for effort condition nor for rinse condition were statistically significant for the extent to which participants felt bored, restless, distracted, fatigued, or engaged (see Table S2). However, it appeared that participants who received the sucrose-sweetened rinse reported feeling less curious than participants who received the sucralose-sweetened rinse, *t*(178)= -2.71, *p* = .007, *d* = -0.41. Other follow-up comparisons were not statistically significant: *t*_unweetened-sucrose_(139)= 1.20, *p* = .23, *d* = .20; *t*_unweetened-sucralose_ (137)= -1.08, *p* = .28, *d* = -.18.

In light of the numerous null findings on the differences between participants’ moods as a function of effort and rinse condition, we find it difficult to have confidence in this single statistically significant result. Therefore, we will not attempt to propose an explanation for it, but rather, include it here in the interest of complete reporting.

**References**

Gjesme, T (1979). Future time orientation as a function of achievement motive, ability, delay of gratification and sex. *Journal of Psychology, 101*, 173-188.

Vohs, KD, Schmeichel, BJ (2003). Self-regulation and the extended now: Controlling the self alters the subjective experience of time. *Journal of Personality and Social Psychology,* *85*, 217–230.
