# Supplementary material for: After a pair of self-control-intensive tasks, sucrose swishing improves subsequent working memory performance
Source: BMC Psychol. 2013 Oct 30;1(1):22. doi: 10.1186/2050-7283-1-22 (PMC4269986; doi:10.1186/2050-7283-1-22)
Supplement: Supplementary file 2 — Additional file 2: Table S2: Results for the three supplemental analysis outcome categories. (DOCX 16 KB) [file 40359_2013_19_MOESM2_ESM.docx]

| *Table S2.* Results for the three supplemental analysis outcome categories. | | | | | |  |  |
| --- | --- | --- | --- | --- | --- | --- | --- |
| Outcome | |  | Main effects | | |  | Interaction effect |
|  |  |  | Effort |  | Rinse |  | Effort x rinse |
| Time estimates | Video |  | *F*(1, 223) = 4.40* |  | *F*(2, 223) = 0.09 |  | *F*(2, 223) = 1.23 |
|  | Essay |  | *F*(1, 214) = 0.26 |  | *F*(2, 214) = 1.98 |  | *F*(2, 214) = 0.78 |
|  | OSPAN |  | *F*(1, 215) = 0.03 |  | *F*(2, 215) = 1.00 |  | *F*(2, 215) = 0.61 |
| Initial task | Video words |  | *F*(1, 229) = 681.28*** |  | *F*(2, 229) = 1.52 |  | *F*(2, 229) = 2.49 |
|  | Drink regularly^†^ |  | *b*_effort_ = 0.49 |  | *b*_sucrose_ = 2.63*** |  | *b*_sucrose*effort_ = 0.51 |
|  |  |  |  |  | *b*_sucralose_ = 2.04** |  | *b*_sucralose*effort_ = 1.01 |
| Mood: | Restless |  | *F*(1, 225) = 0.15 |  | *F*(2, 225) = 1.22 |  | *F*(2, 225) = 1.82 |
|  | Distracted |  | *F*(1, 223) = 0.001 |  | *F*(2, 225) = 0.69 |  | *F*(2, 225) = 0.61 |
|  | Bored |  | *F*(1, 225) = 1.82 |  | *F*(2, 225) = 0.67 |  | *F*(2, 225) = 0.65 |
|  | Fatigued |  | *F*(1, 225) = 0.29 |  | *F*(2, 225) = 0.06 |  | *F*(2, 225) = 0.70 |
|  | Engaged |  | *F*(1, 225) = 0.01 |  | *F*(2, 225) = 0.27 |  | *F*(2, 225) = 0.09 |
|  | Curious |  | *F*(1, 224) = 0.97 |  | *F*(2, 225) = 3.55* |  | *F*(2, 225) = 1.00 |
| *Note.* ****p*<.001; ***p*<.01; **p*< .05. †Indicates that the outcome variable was binary and that it was analyzed using logistic regression. | | | | | | | |
