# Supplementary material for: After a pair of self-control-intensive tasks, sucrose swishing improves subsequent working memory performance
Source: BMC Psychol. 2013 Oct 30;1(1):22. doi: 10.1186/2050-7283-1-22 (PMC4269986; doi:10.1186/2050-7283-1-22)
Supplement: Supplementary file 3 — Additional file 3: Table S1: Cell means, standard deviations, and sample sizes for each combination of effort and rinse conditions. (DOCX 26 KB) [file 40359_2013_19_MOESM3_ESM.docx]

| *Table S1.* Cell means, standard deviations, and sample sizes for each combination of effort and rinse conditions. | | | | | | | | | | | | | | | | | | | | | | | | |
| --- | --- | --- | --- | --- | --- | --- | --- | --- | --- | --- | --- | --- | --- | --- | --- | --- | --- | --- | --- | --- | --- | --- | --- | --- |
| Variable Name |  | High-effort/Sucrose-rinse | | |  | Low-effort/Sucrose-rinse | | |  | High-effort/Sucralose-rinse | | |  | Low-effort/Sucralose-rinse | | |  | High-effort/Unsweetened-rinse | | |  | Low-effort/Unsweetened-rinse | | |
|  |  | M | SD | n |  | M | SD | n |  | Mean | SD | n |  | Mean | SD | n |  | M | SD | n |  | M | SD | n |
| OSPAN component score |  | 0.31 | 1.05 | 45 |  | -0.29 | 0.84 | 48 |  | -0.11 | 1.12 | 45 |  | 0.02 | 0.91 | 47 |  | 0.25 | 1.00 | 26 |  | -0.25 | 1.02 | 24 |
| OSPAN total full sets |  | 7.89 | 2.77 | 45 |  | 6.63 | 2.17 | 48 |  | 7.18 | 3.06 | 45 |  | 7.51 | 2.52 | 47 |  | 7.96 | 2.79 | 26 |  | 6.75 | 2.80 | 24 |
| OSPAN total words |  | 45.51 | 6.11 | 45 |  | 38.73 | 5.81 | 48 |  | 39.80 | 6.80 | 45 |  | 40.40 | 5.20 | 47 |  | 41.54 | 6.60 | 26 |  | 39.38 | 5.90 | 24 |
| OSPAN words in full sets |  | 22.60 | 10.02 | 45 |  | 17.27 | 7.25 | 48 |  | 19.60 | 10.76 | 45 |  | 20.36 | 9.45 | 47 |  | 22.19 | 9.54 | 26 |  | 17.88 | 9.19 | 24 |
| OSPAN longest set |  | 4.04 | 0.90 | 45 |  | 3.54 | 0.74 | 48 |  | 3.58 | 0.84 | 45 |  | 3.72 | 0.71 | 47 |  | 4.00 | 0.69 | 26 |  | 3.46 | 0.93 | 24 |
| OSPAN difficulty |  | 5.62 | 1.72 | 42 |  | 6.51 | 1.18 | 45 |  | 6.52 | 1.73 | 42 |  | 6.73 | 1.58 | 44 |  | 6.52 | 1.27 | 23 |  | 6.73 | 1.03 | 22 |
| OSPAN boringness |  | 3.95 | 2.09 | 42 |  | 4.13 | 2.33 | 45 |  | 4.93 | 2.29 | 42 |  | 4.34 | 2.44 | 44 |  | 4.30 | 2.48 | 23 |  | 4.14 | 1.93 | 22 |
| Video look |  | 2.33 | 1.26 | 45 |  | 7.40 | 1.41 | 48 |  | 2.93 | 1.68 | 45 |  | 7.53 | 1.50 | 47 |  | 2.23 | 1.27 | 26 |  | 7.96 | 1.40 | 24 |
| Video difficulty |  | 4.36 | 2.12 | 45 |  | 4.83 | 1.98 | 48 |  | 3.84 | 2.04 | 45 |  | 4.26 | 2.13 | 47 |  | 3.54 | 2.21 | 26 |  | 3.63 | 2.04 | 24 |
| Essay difficulty |  | 8.36 | 1.21 | 45 |  | 3.71 | 2.09 | 48 |  | 8.51 | 0.84 | 45 |  | 3.53 | 2.18 | 47 |  | 8.69 | 0.62 | 26 |  | 3.96 | 2.63 | 24 |
| Drink pleasantness |  | 4.11 | 2.36 | 45 |  | 4.17 | 2.22 | 48 |  | 4.04 | 2.35 | 45 |  | 5.11 | 2.45 | 47 |  | 6.46 | 1.48 | 26 |  | 6.17 | 1.83 | 24 |
| Drink liking |  | 5.73 | 2.49 | 44 |  | 5.58 | 2.43 | 48 |  | 5.67 | 2.61 | 45 |  | 4.98 | 2.55 | 47 |  | 2.81 | 1.72 | 26 |  | 3.67 | 2.41 | 24 |
| Drink sweetness |  | 7.13 | 1.39 | 45 |  | 7.00 | 1.81 | 48 |  | 6.96 | 1.88 | 45 |  | 7.04 | 1.55 | 47 |  | 3.77 | 2.37 | 26 |  | 4.71 | 3.10 | 24 |
| Drink regularly |  | 0.43 | 0.50 | 42 |  | 0.34 | 0.48 | 47 |  | 0.36 | 0.48 | 42 |  | 0.18 | 0.39 | 45 |  | 0.04 | 0.20 | 25 |  | 0.05 | 0.21 | 22 |
| Pleasant/Unpleasant |  | 7.14 | 6.47 | 44 |  | 5.85 | 6.07 | 47 |  | 6.16 | 6.13 | 43 |  | 5.43 | 7.02 | 47 |  | 6.77 | 5.33 | 26 |  | 6.04 | 7.50 | 24 |
| Active/Calm |  | 13.91 | 3.66 | 44 |  | 13.28 | 3.91 | 47 |  | 12.95 | 3.16 | 43 |  | 12.98 | 3.42 | 47 |  | 12.81 | 3.58 | 26 |  | 14.33 | 3.13 | 24 |
| Bored |  | 2.45 | 0.90 | 44 |  | 2.68 | 0.81 | 47 |  | 2.47 | 0.85 | 43 |  | 2.77 | 0.84 | 47 |  | 2.46 | 0.99 | 26 |  | 2.42 | 0.88 | 24 |
| Restless |  | 2.23 | 0.71 | 44 |  | 1.91 | 0.80 | 47 |  | 2.19 | 0.79 | 43 |  | 2.32 | 0.91 | 47 |  | 2.08 | 0.84 | 26 |  | 2.12 | 0.85 | 24 |
| Distracted |  | 2.25 | 0.87 | 44 |  | 2.09 | 0.89 | 46 |  | 2.31 | 0.87 | 42 |  | 2.30 | 0.93 | 47 |  | 2.23 | 0.95 | 26 |  | 2.42 | 0.93 | 24 |
| Fatigued |  | 2.45 | 1.00 | 44 |  | 2.49 | 1.02 | 47 |  | 2.47 | 0.91 | 43 |  | 2.53 | 1.02 | 47 |  | 2.69 | 0.97 | 26 |  | 2.38 | 0.92 | 24 |
| Engaged |  | 2.68 | 0.80 | 44 |  | 2.66 | 0.79 | 47 |  | 2.77 | 0.90 | 43 |  | 2.70 | 0.83 | 47 |  | 2.62 | 0.57 | 26 |  | 2.67 | 0.56 | 24 |
| Curious |  | 3.20 | 0.85 | 44 |  | 3.13 | 0.88 | 47 |  | 3.35 | 0.69 | 43 |  | 3.59 | 0.58 | 46 |  | 3.27 | 0.78 | 26 |  | 3.42 | 0.78 | 24 |
| OSPAN est-actual time |  | 32.62 | 222.92 | 39 |  | 74.30 | 207.66 | 47 |  | 80.14 | 304.24 | 43 |  | 54.30 | 187.53 | 46 |  | 127.70 | 206.55 | 23 |  | 96.09 | 207.58 | 23 |
| Video est-actual time |  | -45.11 | 103.04 | 44 |  | -47.33 | 115.73 | 46 |  | -27.56 | 138.94 | 45 |  | -60.84 | 114.51 | 44 |  | -3.65 | 116.50 | 26 |  | -71.29 | 126.71 | 24 |
| Essay est-actual time |  | 6.47 | 108.38 | 41 |  | 23.00 | 126.21 | 46 |  | 69.75 | 175.60 | 40 |  | 36.87 | 118.22 | 45 |  | 30.80 | 103.52 | 25 |  | 19.57 | 123.23 | 23 |
| Age |  | 18.66 | 1.16 | 44 |  | 18.38 | 0.87 | 48 |  | 18.49 | 0.87 | 45 |  | 19.04 | 3.00 | 47 |  | 18.62 | 1.13 | 24 |  | 18.38 | 0.71 | 24 |
