# Supplementary material for: After a pair of self-control-intensive tasks, sucrose swishing improves subsequent working memory performance
Source: BMC Psychol. 2013 Oct 30;1(1):22. doi: 10.1186/2050-7283-1-22 (PMC4269986; doi:10.1186/2050-7283-1-22)
Supplement: Supplementary file 4 — Additional file 4: Table S3: Intercorrelations for measures of OSPAN. (DOCX 14 KB) [file 40359_2013_19_MOESM4_ESM.docx]

| *Table S3.* Intercorrelations for measures of OSPAN performance | | | |
| --- | --- | --- | --- |
| OSPAN outcome | Zero-order correlations | | |
|  | 2 | 3 | 4 |
| 1. Total sets | 0.91*** | 0.99*** | 0.76*** |
| 2. Total words | . | 0.90*** | 0.75*** |
| 3. Words in correct sets | . | . | 0.80*** |
| 4. Longest set | . | . | . |
| *Note*. ***p < .001 |  |  |  |
